# Development of a Novel Hematological Malignancy Specific Patient-Reported Outcome Measure (HM-PRO): Content Validity

**DOI:** 10.3389/fphar.2020.00209

**Published:** 2020-03-05

**Authors:** Pushpendra Goswami, Esther N. Oliva, Tatyana Ionova, Roger Else, Jonathan Kell, Adele K. Fielding, Daniel M. Jennings, Marina Karakantza, Saad Al-Ismail, Graham P. Collins, Stewart McConnell, Catherine Langton, Sam Salek

**Affiliations:** ^1^School of Life and Medical Sciences, University of Hertfordshire, Hatfield, United Kingdom; ^2^Haematology Unit, Grande Ospedale Metropolitano, Reggio Calabria, Italy; ^3^St. Petersburg State University Medical Center and Multinational Centre for Quality of Life Research, Saint Petersburg, Russia; ^4^Patient Research Partner, Milton Keynes, United Kingdom; ^5^Cardiff and Vale University Health Board, Cardiff, United Kingdom; ^6^Cancer Institute, University College London, London, United Kingdom; ^7^Royal Surrey NHS Foundation Trust, Guildford, United Kingdom; ^8^Leeds Teaching Hospitals NHS Trust, Leeds, United Kingdom; ^9^Singleton Hospital, ABM University Health Board, Swansea, United Kingdom; ^10^Oxford University Hospital NHS Foundation Trust, Oxford, United Kingdom

**Keywords:** hematological malignancy, HM-PRO, quality of life, symptoms, content validity, clinical practice, clinical research

## Abstract

**Background:**

The quality of life of patients at all stages of hematological malignancy is greatly affected by the disease and its treatment. There is a wide range of health-related quality of life (HRQoL) issues important to these patients. Any new instrument developed to measure HRQoL of such patients should be content valid, i.e., the items should be comprehensively relevant to the patients and their health condition. The aim of the present study was to examine content validity of a hematological malignancy specific patient reported outcome measure (HM-PRO) developed for use in routine clinical practice.

**Methods:**

Following literature review and semi-structured interviews, the generated themes and sub-themes were discussed to develop the prototype version of the HM-PRO. A 4-step approach was used for content validation: initial testing and cognitive interviewing; item rating; content validity panel meeting; final field testing and cognitive interviewing. Additional questions related to patients’ perception of recall period and preferred sentence structure (i.e., question or statement) of the items were also asked during cognitive interviews.

**Results:**

The content analysis of 129 transcribed semi-structured interviews resulted in the prototype version of the instrument consisting of 58 items grouped into two parts: Part A (impact/HRQoL – 34 items) and Part B (signs and symptoms – 24 items). The initial testing showed intra-class correlation coefficient (ICC) of >0.8 for both Part A and Part B. Item rating for language clarity, completeness, relevance, and response scale by experts and patients showed content validity index for scales average >0.8 for both Part A and Part B, except 0.64 for relevance for Part A by the patient panel. The final testing of the revised version of the instrument showed the Cronbach’s alpha value of 0.91 for Part A and 0.76 for Part B, suggesting high internal consistency, and ICC of 0.91 for Part A and 0.76 for Part B. The recall period of “today” for Part-A and “last 3 days” for Part-B were the patients’ preferred “recall period.” Furthermore, the patients expressed preference to the HM-PRO items as statements.

**Conclusion:**

The findings of this study confirm that the HM-PRO possesses a strong content validity, includes all the issues important to patients and is easy to read, understand and respond to spontaneously.

## Introduction

Hematological malignancies (HM) are type of cancers which affect the blood, bone marrow, lymphatic system, and production and function of the blood cells (American Society of Haematology, 2018). They include leukemias, lymphomas and myeloma. In the United Kingdom, 30,000 patients are diagnosed each year and the relative 5-year survival is 69.1%, bearing in mind that some leukemias’ prognosis is not as good as others (Department of Health Sciences, 2017). The WHO defines the primary objectives of a cancer diagnosis as cure, prolongation of life, and improvement in quality of life (QoL) (World Health Organization [WHO], 2017). Several studies have previously reported that HM has a high impact on patients’ HRQoL (Persson et al., 2001; Holzner et al., 2004; Santos et al., 2006; Mols et al., 2007; Shanafelt et al., 2007; Strasser-Weippl and Ludwig, 2008; Johnsen et al., 2009). A recent systematic review reported that there are 30 HRQoL instruments currently used in hematology and none of these instruments captures all the issues important to these patients (Goswami et al., 2019). Furthermore, this review also reports that a barrier to using patient-reported outcomes (PRO) in clinical practice is the diversity of such instruments. The oncology generic instruments like EORTC QLQ-C30, FACT-G (Stead et al., 1999; Cella et al., 2012) and their disease specific modules, such as EORTC QLQ-MY20, EORTC QLQ-CML24, FACT-Leu (Cocks et al., 2007; Kontodimopoulos et al., 2012; Jones et al., 2013; Efficace et al., 2014; Espinoza-Zamora et al., 2015) which are currently used have been developed and validated to be used in clinical trials. Only one instrument, MYPOS has been recently developed and validated to be used in clinical practice specific for myeloma and follicular lymphoma patients (Osborne et al., 2015; Davies et al., 2017), and therefore cannot be applied to other HM. Among the identified 30 HRQoL instruments, only partial evidence on the content validity was identified for EORTC QLQ-MY24, FACT-Leu, EORTC Leu, FACT-Lym, QoL-E (Goswami et al., 2019). Up to 18% of the patients resported missing items for EORTC QLQ-MY24 (Goswami et al., 2019). Considering the high number of instruments which are currently used in hematology and the gaps in their measurement properties, there is a need for a generic instrument possessing strong validity for use in clinical practice that could be applied to all HMs (Goswami et al., 2019).

Content validity is the ability of a patient-reported outcome measure (PROM) to assess concepts that it purports to measure (Patrick et al., 2011). It is the first “proof of concept” that an instrument’s content is connected to the construct being measured (Streiner and Normal, 2003). The importance of content validity early in developing PRO measures has been emphasized by both the United States FDA and the EMA (Patrick et al., 2011) and it is postulated that without such evidence the interpretability of the scores may not be established (Haynes et al., 1995). The FDA has recently reported the inability of static questionnaires to ensure content validity in trials of new medicines (Kluetz et al., 2016). In the absence of a PRO measure which could be used for measuring HRQoL and disease symptoms of patients with different HM in daily clinical practice, a new hematological malignancy specific patients-reported outcomes instrument, HM-PRO, has been developed and being validated for such purpose. The aim of this study was to examine content and face validity of the HM-PRO for use in routine clinical practice and research.

## Materials and Methods

The conceptualization, development and the validation of the new instrument was carried out between March 2014 to September 2018. For the first step, the systematic review informed the hypothesized conceptual framework based on the issues identified in the literature (Goswami et al., 2019). Following the systematic literature review in-depth semi-structured qualitative face-to-face interviews were carried out between October 2015 and May 2016, a process by which concepts (i.e., symptoms and impacts) that are important to patients emerged through the use of open-ended questions. The patient interview guide was developed with the help of a patient research partner and pilot tested. A total of 129 patients with different HM’s were recruited from six secondary care hospitals across England and Wales (Goswami et al., 2016). The content analysis of the transcribed interviews was carried out using the NVivo 11, qualitative data analysis software, to identify the themes and sub-themes reported important by the HM patients (Goswami et al., 2017; Velikova et al., 2017). The analysis was validated independently by another researcher. The item pool was then used to develop the first draft of the instrument. This study investigated the content validity and face validity of the HM-PRO which were carried out between September and November 2016 in the United Kingdom.

### Study Design and Recruitment

A mixed method research approach was used to develop a psychometrically sound HRQoL instrument in HM. Adult patients were included in the study if they were diagnosed with HM as per most recent WHO classification, at any state of the disease (stable, progressing, and remission), at any stage of the treatment and were able to read and write in English. A purposive sample was chosen with variation of the type of HM and disease state. In-patients and out-patients were screened by the clinical team for eligible patients and those selected were approached by a member of the clinical care team and those agreed were enrolled onto the study.

### Instrument Construction: Data Definition Panel Meeting

Following the qualitative interviews and their transformation into themes and sub-themes, a 2-day “data definition panel meeting” was organized. The main objectives of the meeting were: to discuss and select the items to be included in the prototype version of the instrument; and to discuss and reach consensus regarding the recall period, presentation of the selected items either as a question or a statement, response options, phraseology of the items, and the general structure of the instrument. The members of the data definition panel meeting included four researchers (specialized in the development and application of PRO instruments), two hematologists (one from secondary care hospital in the United Kingdom and the other from Italy), a representative of hematology patient advocacy group from Germany and a hematology patient research partner (a member of the research team). The members of the panel were those who were involved in the initial conceptualization of the development of the instrument. All the decisions were made by verbal consensus among the panel members. This was an important step to make sure to involve different stakeholders and their perspective in the development of the new instrument, keeping patients’ perspective at the center of the discussion. All the items with a prevalence of more than 5% were discussed individually and those with a prevalence of less than 5% were reviewed if it related to any specific type of HM.

### Content and Face Validation

**FIGURE 1:**
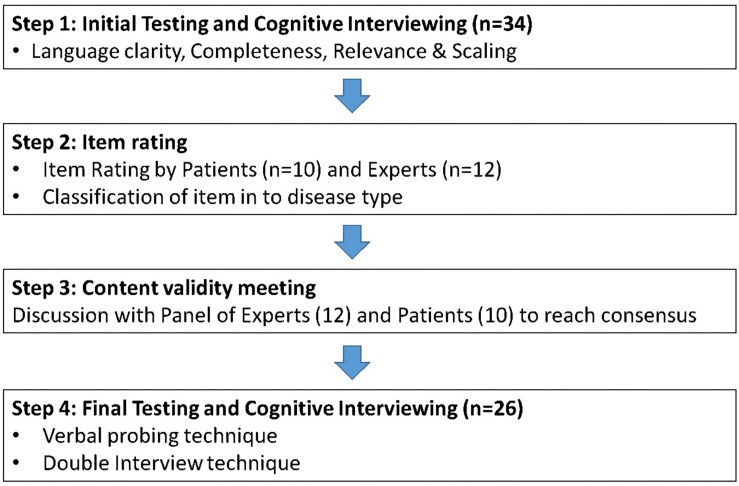
Four-step approach for Content and Face validation of the HM-PRO.

The data definition panel meeting resulted in development of the first version of the instrument. The testing of the new instrument to establish its content and face validity was carried out using a four-step process. The study was carried out between August and November 2017 at four secondary care hospitals in England and Wales. The content validity of the instrument is generally determined using the viewpoints of a panel of experts. In the current study, we specifically emphasized incorporation of patients’ perception in the validation process along with expert opinion. The four-step validation process involved: initial testing and cognitive interviewing; item rating; content validity meeting; and final testing and cognitive interviewing (Figure 1). “Question and answer model” reported by Collins (2003) was used during the cognitive interviews to pre-test the instrument (Collins, 2003).

#### Step 1: Initial Testing and Cognitive Interviewing

In order to ensure that the items in the instrument are easily understood, unambiguous, jargon-free and are relevant to patients and the construct being measured, it is advised to pre-test it in the target population (Streiner and Normal, 2003). In the initial testing phase, patients with different HM were asked to complete the instrument at two secondary care hospitals. Patients were also asked to answer a series of additional questions (i.e., were the questions easy to read; were the statement easy to understand; were you able to respond to the statements spontaneously; would you be willing to complete this questionnaire every time you visit the clinic; have all the aspects important to you been covered; is there anything you would like to delete; and is there anything you would like to add) with respect to four essential relevance, applicability and practicality criteria: language clarity; completeness; relevance; and response scales (Table 1). Patients were randomly selected for cognitive interviewing. Both double interviewing technique and verbal probing technique were used by the interviewer (Streiner and Normal, 2003). The interviews were audio recorded, transcribed verbatim and content analysis was performed using NVivo 11, a qualitative analysis software. The probes defined by Collins (2003) were used during the interviews (Collins, 2003). Patients were also asked about the preferred recall period for the items after the concept was explained to them: “what would be the appropriate recall period from their perspective which could allow them to respond to an item spontaneously and represent the true state of their HRQoL.” The inter-rater agreement was assessed by calculating ICC. ICC is a type of reliability index that reflects both agreement, as well degree of correlation between measurements (Koo and Li, 2016). In addition, internal consistency reliability was assessed using Cronbach’s alpha.

**TABLE 1:** Criteria for relevance, applicability and practicality of the HRQoL instrument.

**Criteria**	**Definition**
Language clarity	Wording should be clear, understandable, simple, unambiguous and jargon free. Should be understood by someone with reading ability of a 12 year old (Streiner and Normal, 2003).
Completeness	Sentences should be complete, unbroken and should end appropriately (Guyatt et al., 1993).
Relevance	Each item should reflect as HRQoL and symptoms, important to patients with hematological malignancy, and also relevant to the construct being measured (Leidy et al., 1999).
Scaling response	The choice of response option must fit the items and be appropriate to the construct being measured (DeVellis, 2016)

#### Step 2: Item Rating

In the item rating phase, the investigator and a hematology clinic nurse at each participating NHS site were approached inviting them to participate in the Item Rating phase. The investigators were also asked to nominate a patient from their hematology clinic list who would be willing to take part in the “content validity” study. In addition, four PRO experts were invited to take part. A total of 10 HM patients and 12 experts (4 hematologists, 4 PRO methodologists and 4 hematology clinic nurses) rated each item of the first draft (prototype) of the instrument for its language clarity, completeness, relevance and scaling response on a 4-point Likert scale of “strongly disagree to strongly agree.” The process was carried out using an excel file which was sent to each rater via email. The level of agreement between the raters was calculated using content validity index (CVI) (Shi et al., 2012). The CVI was calculated at both the item and scale levels. The minimum acceptable interrater agreement at the scale level was set at 0.80 and at 0.79 for the item level (Zamanzadeh et al., 2015). In order to maintain the confidence in selecting the most important and essential content in the instrument, the experts were asked to classify each item as important as per disease type (Zamanzadeh et al., 2015). The content validity ratio (CVR) was calculated and the CVR >0.99 was accepted as per Lawshe’s (1975) table depending on the number of panelists (Lawshe, 1975). Additional space was provided for feedback and suggestions for each item.

#### Step 3: Content Validity Meeting (CVM)

Following the initial testing and rating of the items in the second stage the results were discussed during a content validity meeting involving two different panels of experts and patients. A 2-round Delphi method for consensus was used. The main objectives of the content validity meeting were:

(a) to resolve the issues or any discrepancies observed during initial testing and item rating;
(b) to reach consensus between the panels of experts and patients; and
(c) incorporate the panelists’ views in order to support development of the revised version of the instrument.

#### Step 4: Final Testing and Cognitive Interviewing

Finally, the revised version developed during the content validity meeting was again tested in order to examine the patients’ understanding of the items in the instrument. Both double interview and verbal probing techniques were incorporated in the face-to-face cognitive interviewing. The interviews were audio recorded, transcribed verbatim and content analysis was performed using NVivo 11, a qualitative analysis software. Patients were asked to read the HM-PRO items aloud and then were asked questions such as “how did you come to that answer” or “what do you understand by the term…” (Foddy, 1993; Streiner and Normal, 2003). In addition, patients were asked whether they preferred sentence structure of the items to be represented as statements or as questions.

## Results

### Instrument Construction: Data Definition Panel Meeting

Following the content analysis, item generation was carried out by the data definition panel members who discussed the most prevalent items on the basis of their clinical significance, as well as on the basis of their importance from a patient’s perspective to be included in the first version of the instrument, the prototype. The patient research partner and the representative of the patient advocacy group expressed the patients’ and their own perspective. The consensus was reached by discussion among the panel members. In total, 58 Items including QoL issues as well as signs and symptoms were selected to be included in the prototype instrument. The paper-based instrument was named “HM-PRO.”

### Instrument Design

As the HM-PRO has been developed for use in routine clinical practice, consensus was reached by the panel to develop the instrument as a composite measure. Thus, it consists of two independent scales: Part A – measuring “impact” i.e., quality of life; and Part B – measuring “signs and symptoms.” The panel agreed that a composite measure would have a greater potential to help a clinician not only in identifying issues important to a patient, but also should assist in diagnosis and identifying changes in the disease state based on the outcome of the “signs and symptoms” scale. Based on the conceptual framework developed from the literature search and result of the qualitative phase, Part A was developed with five domains comprising 33 items. The five domains include: physical well-being; social and family well-being; emotional well-being; eating and drinking habits; and treatment and healthcare services. Whereas, Part B was developed as a single domain of “signs and symptoms” consisting of 25 items (Supplementary Appendix S1).

The panel also resolved to present the items as “statements” rather than as “questions.” A 5-point intensity Likert scale of “Not at all” to “A lot” was chosen as the scaling response options for Part A, and a 4-point severity Likert scale of “Not at all” to “Severe” was chosen for Part B.

### Content and Face Validation

A total of 60 patients were recruited into a 4-step content and face validity exercise of whom 34 took part in the initial testing (step 1) and then a cohort of 26 different patients in the final testing (step 4). None of the patients were followed up or re-recruited into the study.

#### Step 1: Initial Testing and Cognitive Interviewing

Thirty-four patients (Male = 19; mean age = 67.4 years; SD = ± 14.9 years; median age = 71.1 years, age range = 25–91 years; and IQR = 10.9 years) with mean time since diagnosis of 3.7 years (SD = ± 5.6 years; median years = 1.7 years; range = 51 days to 26 years; and IQR = 4.3 years) were recruited into the first step of the study (Table 2). Ten (29.4%) patients had other comorbidities, of whom one had other types of cancer. The patients were recruited from six secondary care hospitals across England and Wales. All 34 patients completed the first draft of the instrument. The Cronbach’s alpha for Part A was 0.95 and Part B was 0.85, suggesting high internal consistency.

**TABLE 2:** Demographics characteristics of the study participants – initial testing (*n* = 34) and final testing (*n* = 26).

		**Initial testing (*n* = 34)**		**Final testing (*n* = 26)**	
		**Mean (± SD)**	**Range**	**Mean (± SD)**	**Range**
Age (Years)		67.4 (± 14.9)	25–91	59.1 (± 18.2)	21–84
Time since Diagnosis		3.7 (± 5.6)	0.13–26	6.4 (± 7.2)	0.03–22
(years)					

		***n***	**%**	***n***	**%**

Sex	Male	19	55.9	17	65.4
	Female	15	44.1	9	34.6
Ethnic Origin	White	33	97.1	25	96.2
	Black British	1	2.9	1	3.8
Disease Type	AML	9	26.5	1	3.8
	ALL	0	0.0	1	3.8
	CLL	2	5.9	0	0
	MM	13	38.2	4	15.4
	ANHL	3	8.8	5	19.2
	INHL	4	11.8	6	23.1
	CML	0	0.0	2	7.7
	MPN	1	2.9	1	3.8
	MDS	1	2.9	2	7.7
	HL	1	2.9	4	15.4
Disease State	Stable	7	11.7	4	15.4
	Remission	12	20.0	14	53.8
	Progressing	5	8.3	8	30.8
	Unknown	10	16.7	–	–

*n, number of patients recruited; SD, standard deviation; AML, acute myeloid leukemia; ALL, acute lymphoid leukemia; CLL, chronic lymphoid leukemia; MM, multiple myeloma; ANHL, aggressive non-Hodgkin lymphoma; INHL, indolent non-Hodgkin lymphoma, CML. Chronic myeloid leukemia; MPN, myeloproliferative neoplasm; MDS, myelodysplastic syndromes; HL, Hodgkin lymphoma.*

The relative reliability, calculated using the ICC, was 0.95 (95% confidence interval, CI = 0.91 to 0.98) for Part A, and ICC of 0.85 (95% CI = 0.75 to 0.92) for Part B. The highest missing item was “work/studies,” followed by items for sex life, medication management and sports. Nine patients wrote “Not Applicable” next to the response options for 13 items, instead of selecting “Not at all” response option. The result of the additional questions asked from the patients are presented in Table 3. One patient commented that “*Part A needs to be reworked if older retired patients are completing the questionnaire.*” None of the patients wanted to delete any item except one who suggested to delete item 24 for bowel problems from Part B, because she felt it is covered by other items i.e., constipation and diarrhea. Two patients suggested including items related to panic attacks, anxiety attacks, medical information and mood state for depression screening.

**TABLE 3:** Cognitive interviewing – practicality and applicability of the HM-PRO: initial (*n* = 34) and final testing (*n* = 26).

		**Initial testing with**		**Final testing with**	
		**first version of the**		**second version of the**	
		**instrument (*n* = 34)**		**instrument (*n* = 26)**	
**No**	**Questions**	**Yes, n (%)**	**No, n (%)**	**Yes, n (%)**	**No, n (%)**
1	Were the questions easy to read?	33 (97)	1 (3)	25 (96)	1 (4)
2	Were the statements easy to understand?	30 (88)	4 (12)	25 (96)	1 (4)
3	Were you able to respond to the statements spontaneously?	33 (97)	1 (3)	24 (92)	2 (8)
4	Would you be willing to complete this questionnaire every time you visit the clinic?	34 (100)	0 (0)	23 (88)	3 (12)
5	Have all the aspects important to you been covered?	32 (94)	2 (6)	23 (88)	3 (12)
6	Is there anything you would like to delete?	1 (3)	33 (97)	0 (0)	26 (100)
7	Is there anything you would like to add?	2 (6)	32 (94)	5 (19)	21 (81)

With respect to cognitive interviews, eight patients were randomly selected and interviewed by PG. Four items were identified which were difficult for the patients to understand: “I am troubled with time spent in hospital”; “I worry about the treatment”; “I do not feel confident”; and “I am satisfied with healthcare services.” During the interviews, four patients mentioned having difficulty in differentiating the following items: tiredness and fatigue; holidays and traveling; depression and distress; back pain, body pain and bone pain; and bowel problems, constipation and diarrhea’. When asked about the choice of recall period, the majority of the patients preferred “real time” (i.e., today – at the moment) as the chosen recall period for Part A, and “last 3 days” for Part B (Table 4). When the patients were asked about the recall period for symptoms, one commented that “*past 3 days is good enough because for example – while answering about pain, I was thinking about how many days I was in pain, and whether I was in pain the whole day or not.” When the* patients were asked about their views concerning the recall period for Part A (impact), one commented “*I think using “today” is better, I had a hectic week last week, I went to a funeral, I had other things, I was a bit anxious.*”

**TABLE 4:** Study patients’ perspective of recall period (*n* = 34).

	**Number**	**%**
**Recall period for part A (*n* = 34)**		
Last 1 week	8	24
3 Days	2	6
Today	22	65
Unable to understand	2	6
**Recall period for part B (*n* = 34)**		
Last 3 days	18	53
Last 1 week	7	21
More than a week	2	6
Today (real time)	2	6
Unable to understand	5	15

#### Step 2: Item Rating

All the items in Part A and Part B of the HM-PRO were rated by 12 experts and 10 patients for its language clarity, relevance, completeness, and scaling. The I-CVI for language clarity of Part A ranged from 0.63 to 1.00 with 16/33 items reaching I-CVI of 1.00, for Part B the I-CVI was >0.7 for all the items for language clarity with 9/25 items reaching CVI of 1.00 (Tables 5, 6). The I-CVI for completeness of the items for Part A and Part B was >0.75 for all the items except two items from Part A and one from part B. All the raters agreed on the relevance of the items in both Part A and Part B with I-CVI >0.7 for all the items and universal agreement of 23/33 and 21/25 items from Part A and B, respectively. The scaling response had a scale content validity index (S-CVI) of >0.75 for all the items except for one item in Part B. The CVI at scale level was >0.8 for language clarity, completeness, relevance, and scaling. Finally, CVR was calculated for all the items which were reported relevant to each HM by the experts (Table 7).

**TABLE 5:** Content validity – agreement between members of the expert panel.

	**Language clarity**	**Completeness**	**Relevance**	**Scaling**
**Part A**				
I-CVI >0.78	25 of 33 Items	21 of 33 Items	32 of 33 Items	31 of 33 Items
S-CVI/Ave	0.89	0.84	0.96	0.86
Total agreement (%)	16.00 (48.48%)	6.00 (18.18%)	23.00 (69.70%)	0.00 (0%)
**Part B**				
I-CVI >0.78	18 of 25 Items	16 of 25 Items	24 of 25 Items	24 of 25 Items
S-CVI/Ave	0.89	0.85	0.98	0.96
Total agreement (%)	9.00 (36%)	5 (20%)	21.00 (84%)	17.00 (68%)

*I-CVI = Item – content validity index (Acceptable – ≥0.78) (Polit and Beck, 2006). S-CVI/Ave = scale – content validity index average (Acceptable – ≥0.8) (LindseyDavis, 1992; Grant and Davis, 1997; Polit and Beck, 2006). Total agreement- I-CVI = 1. Universal agreement for all 4 criteria for PART A = 4 of 33 Items. Universal agreement for all 4 criteria for PART B = 6 of 25 Items.*

**TABLE 6:** Content validity – agreement between members of the patient panel.

	**Language clarity**	**Completeness**	**Relevance**	**Scaling**
**Part A**				
I-CVI >0.78	30 of 33 Items	30 of 33 Items	30 of 33 Items	33 of 33 Items
S-CVI/Ave	0.87	0.87	0.64	0.94
Total agreement (%)	12 (36.36%)	11 (33.33%)	21 (63.64%)	21 (63.64%)
**Part B**				
I-CVI >0.78	23 of 25 Items	24 of 25 Items	24 of 25 Items	20 of 25 Items
S-CVI/Ave	0.95	0.93	0.95	0.90
Total agreement (%)	19 (76%)	17 (68%)	18 (72%)	15 (60%)

*Universal agreement for all 4 criteria for PART A = 4 of 33 Items. Universal agreement for all 4 criteria for PART B = 8 of 25 Items.*

**TABLE 7:** Content validity ratio across HM.

		**AML**	**CML**	**ALL**	**CLL**	**MM**	**ANHL**	**INHL**	**HL**	**MPN**	**MDS**
No. of items with CVR >0.99*	**PART A**	27	11	26	14	21	22	16	22	8	18
	**PART B**	18	5	19	15	15	20	14	19	7	7

*Acceptable criteria as per Lawshe’s table for 5 panelists (Lawshe, 1975).

#### Step 3: Content Validity Meeting

A panel of 12 experts and 10 patients who rated items in the previous steps were invited to attend the content validity meeting (CVM) and all the comments and suggestions were discussed to reach consensus. A 2-round Delphi method for consensus was used. The item rating results provided the first round and the verbal discussion in the content validity meeting was the second round. There was a universal agreement between panel of experts and patients for 23/33 items in Part A and 18/25 items in Part B. No amendments were made to these items and were included as they were in the second version of the instrument. The experts agreed on moving the item related to weight change from Part B to Part A as an impact and rephrased it from “My weight has changed” to “I am concerned about my weight change.” The item related to sports was rephrased from “I have difficulty doing sports” to “I have difficulty with physical activity/sports,” this was based on patients comment that gardening can be a physical activity but is not a sport. The “expert panel” suggested to remove the item related to bowel problems, but the “patient panel” insisted to keep the item as they considered it different from diarrhea and constipation. The remaining items had minor amendments related to grammar or providing example (s) for selected items. For example, “transfusions are burden for me (e.g., blood, platelets).” With respect to the response options, considering the mean age at diagnosis of 70.8 years for HM (Department of Health Sciences, 2017), some of the items were not applicable to the older patients, hence an additional response option “Not Applicable” was included in the impact part of the instrument following consensus reached between the expert and patient panels. The content validity meeting resulted in the development of the second version of the HM-PRO with 34 items for “impact” (Part A) and 24 items for “signs and symptoms” (Part B).

#### Step 4: Final Testing and Cognitive Interviewing

In the final step 26 different patients (Male = 17; mean age = 59.1 years; SD = ± 18.2; median age = 64.7; age range = 21–84 years; and IQR = 19.21 years) with mean time since diagnosis of 6.4 years (SD = ± 7.2 years; median = 3.75; range = 14 days to 22 years; and IQR = 9.4 years) were recruited to test the second version of the instrument (Table 2). All 26 patients completed the HM-PRO, answered the additional practicality/applicability questions and were subsequently interviewed. The Cronbach’s alpha for the revised version was 0.91 for Part A and 0.76 for Part B, suggesting high internal consistency. The response rate was improved after introducing the “not applicable” as a response option and there were only two missing responses in Part A, and two in Part B. The ICC of the revised version of the instrument was 0.91 (95% CI ranging between 0.85 and 95) for Part A and 0.76 (95% CI ranging between 0.61 and 0.88) for Part B. With respect to the additional questions, none of the patients suggested deleting any items and 19 (81%) patients did not want to add any item (Table 3). Those who suggested additional items had very low prevalence and hence were not included in the instrument. All 26 patients were interviewed and asked to explain the reason or rationale of their responses. All the patients were able to understand the items and statements in the way they were intended, confirming the face validity of the items and the scale. The patients were able to differentiate between the items like “I have difficulty traveling (e.g., bus, train, flight and car)” and “I have difficulty going on holiday” after the examples were included in the item as a result of the content validity meeting. The patients were also asked if they preferred the items to be represented as “statements” or “questions”: 15 (57.69%) preferred the items as “statements,” 9 (34.61%) as “questions,” whereas 2 (7.69%) were indifferent. One of the patients said “*when you ask the question you can leave it open ended and not be specific. So, I think statement is better*” and another patient said “*Probably I won’t be spontaneous if it’s a question. A statement is straightforward.*”

## Discussion

The psychometric properties of an instrument rely on its ability to measure what it purports to measure and whether it is capturing all the relevant information to the patient, hence the link between the content of the instrument and its underlying construct is of great importance (Wynd et al., 2003). Furthermore, major regulatory authorities such as FDA, EMA, Therapeutic Good Administration (TGA) Australia, etc. have put special emphasis on the content validation of newly developed PROMs (Patrick et al., 2011). This study proposed to establish the content and face validity of the first HM-specific PRO instrument (HM-PRO) for use in routine clinical practice as well as in research. The study involved a four-step approach for content validation, incorporating comments and suggestions from all relevant stakeholders in hematology and, most importantly, patients themselves. Patients with different types of HM and in different disease states may have different issues important to them, hence a good mix of patients with different disease types and disease states were recruited. The study participants were asked questions, in a comprehensive manner, related to the content and face validity to incorporate their perspective in the validation process. The result of the initial testing and cognitive interviewing has indicated that the items of the first version (prototype) of the HM-PRO were easy to understand, easy to respond to and relevant to them. Some patients raised concerns about not being able to respond to the issues related to sex life, work life and sports as they were not applicable to them because of their age. The CVI calculated at the item and scale level showed strong agreement between experts and patients for language clarity, completeness, relevance, and scaling. During the content validity meeting the panels further discussed the suggestion made by the patients and experts and provided further information on the structure and organization of the instrument. A general consensus was reached to include “not applicable” as a response option in the HM-PRO scaling and some minor changes were made to the items without removing any item from the scale. The final testing of the second version of the HM-PRO, had similar outcome confirming that the items are easy to understand and respond to and that they cover all the issues relevant to them. Few patients suggested including one or two items, but they were dismissed due to low prevalence. One of the major outcomes of this study was being able to understand patient preferences for “recall period” and format of the items either as “statement” or “question.” Patients preferred “today” i.e., real time as the recall period for impact (Part A) and “last 3 days” for signs and symptoms (Part B), this ensures the absence of underestimation or overestimation of the impact due to recall biases (Norquist et al., 2012). Sentence structure for the Items as “statement” was preferred over “question,” for being less open ended. The verbal probing technique for cognitive interviews (using general, comprehension, retrieval, judgment, response and acceptability as the probes (Collins, 2003) confirmed that the patients were thinking in the direction expected, understanding the statements and the terms as expected and were able to rephrase the statements keeping the same meaning as intended. Thus, we demonstrated that the newly developed PRO measure for HM patients exhibits satisfactory content validity and face validity and is informative tool to identify the impact of the disease and its treatment on patients’ HRQoL as well as signs and symptoms in this heterogeneous patient population in routine hematological practice.

As identified by the systematic review, there are a number of gaps in the currently used PROs in hematology with respect to their content as well as their measurement properties (Goswami et al., 2019). The important aspects of HRQoL such as “living with uncertainty, eating and drinking habits, body image/appearance, burden of disease and the treatment” are not captured by the existing instruments. Furthermore, all such instruments have been developed without using modern and sophisticated techniques which are employed to improve the precision of a measurement instrument. The HM-PRO has been developed with an aim to fill in the existing gaps using both traditional classical test theory as well as advanced item response theory. Furthermore, patient-centric approach has been incorporated in the development of the HM-PRO as per the 2009 FDA PRO development guidelines.

The concept of HRQoL is very subjective. Every patient has different perspective toward their own HRQoL. What is important to patients in terms of HRQoL issues may differ from individual to individual. A traveller would be affected more if he/she cannot travel due to their condition compared to a sports person who would be affected more if he/she cannot play any sports because of their condition. Although, the medicines taken during the treatment might have the same mechanism of action, but it may affect each patient differently. Some have high tolerance to pain whereas others may find it extremely difficult to cope with slightest pain. Some patients cope and respond to the chemotherapy very well, whereas, others might experience a lot of side effects (Cancer Council Victoria, 2019). Furthermore, there are different aspects of patients’ HRQoL which are affected significantly, but patients are hesitant to discuss them with their clinicians. For example, it is known that HMs and their treatment may affect a patient’s sexual life (McGrath, 2012), but the issue being very personal to the patients is not usually discussed during consultation. Therefore, HM-PRO can be used to facilitate the discussion between the patient and clinician on individual basis to deliver a patient-centered care.

The HM-PRO can also be used for the purpose of focusing on a specific functional area for which the patient is mostly affected. For example, if the disease or the treatment is affecting the patient psychologically, the HM-PRO will be able to detect this by displaying high scores on the “emotional behavior” domain of Part A. If this individual domain is scored higher in subsequent two-three evaluations then patients might be referred to the mental health services or even the hematologist can try to speak to the patient to identify what is affecting his/her psychological well-being and provide guidance accordingly. The clinicians have the tendency to trust their own *ad hoc* assessment of a patient’s HRQoL, but they are not always able to do this accurately (Lohr and Zebrack, 2008). Therefore, the HM-PRO might be useful to identify specific functional issues on individual basis early in the course of the disease and treatment.

Furthermore, the HM-PRO can be used as a patient management tool for monitoring a patient’s condition over time on an individual basis. The information collected through the use of the HM-PRO could be used together with the clinical information collected through other diagnostic test and then treatment or patient monitoring strategies can be developed based on the individual patient needs. Further, the impact on HRQoL can be monitored over time to see how the patient is responding to the treatment.

## Strengths and Limitations

Some limitation of the content validity study should be noted. Item rating and classifying the issues specific to different HM by experts is subjective and therefore open to bias, since their ratings are based on their experience from their own individual practice. Hence, the CVR might vary with different panel of experts. However, four hematologists and four clinic nurses with expertise in different HM, were involved in the process to minimize such bias. Usually, the PRO developers carryout cognitive debriefing only once and make the changes according to the findings and do not test the new version of the instrument. This study carried out cognitive debriefing interviews before and after the changes were made, hence making sure that the HM-PRO has strong content and face validity and the items have language clarity, completeness, are relevant to patients and the response options allow patients to respond spontaneously. This should be considered as one of the cardinal strengths of the study, minimizing person and item response variability. In addition, the results of Part B (signs and symptoms) of the HM-PRO may reflect a side effect and/or a symptom of the disease state. However, patients will not be able to report whether “I have hair loss” (for example) related to a side effect of treatment or a symptom? This, of course, will become evident with further probing during the consultation.

## Conclusion

The outcome of this study clearly indicates that the HM-PRO is content valid across different HM and measures what it is supposed to measure. Its focus and emphasis are fit for purpose demonstrating relevance to the target population. All the patients expressed interest in completing HM-PRO every time they visit the hematology clinic of their care provider hospital.

## Data Availability Statement

The datasets generated for this study are available on request to the corresponding authors.

## Ethics Statement

Multicenter ethics approval was obtained from the NRES South West Bristol, United Kingdom (ref 14/SW/0033) followed by individual R&D approvals from all the participating centers. Signed informed consent was obtained from all the study participants.

## Author Contributions

PG collected the data, carried out the qualitative interviews and their transcription, developed the analysis policy, liaised with hospitals for patient recruitment, analyzed the data, interpreted results, and wrote the first draft of the manuscript. RE contributed to the data collection as a patient research partner, validated the transcribed interviews, and reviewed the draft manuscript. SS generated the original idea, developed the study protocol, supervised the study, liaised with study centers as part of patient recruitment, developed the analysis policy, interpreted the results, and reviewed the draft manuscript. EO and TI contributed to the development of the study protocol, interpreted the results, and reviewed the draft manuscript. JK, AF, DJ, MK, SA-I, GC, SM, and CL contributed to the patient recruitment from their respective center and reviewed the draft manuscript. In addition, PG, EO, TI, RE, JK, and SS took part as panel members in the data definition meeting for the item generation phase; and PG, EO, AF, SA-I, DJ, SM, and SS as expert panel members in the content validity meeting.

## Conflict of Interest

The authors declare that the research was conducted in the absence of any commercial or financial relationships that could be construed as a potential conflict of interest.

## Abbreviations

ALL: acute lymphoid leukemia
AML: acute myeloid leukemia
ANHL: aggressive non-Hodgkin lymphoma
CLL: chronic lymphoid leukemia
CML: chronic myeloid leukemia
CVI: content validity index
CVR: content validity ratio
EMA: european medicines agency
EORTC QLQ C-30: european organization for the research and treatment of cancer quality of life questionnaire -core 30
EORTC QLQ MY-20: european organization for the research and treatment of cancer quality of life questionnaire – myeloma 20
EORTC QLQ-CML24: european organization for the research and treatment of cancer quality of life questionnaire -chronic myeloid leukaemia-24
FACT-G: functional assessment of cancer therapy: general
FACT-Leu: functional assessment of cancer therapy: leukemia
FDA: food and drug administration
HL: hodgkin lymphoma
HM: hematological malignancy
HM-PRO: hematological malignancy patient-reported outcome measure
HRQoL: health-related quality of life
ICC: intra-class correlation
I-CVI: item-content validity index
INHL: indolent non-Hodgkin lymphoma.
