# Supplementary material for: Development of a Novel Hematological Malignancy Specific Patient-Reported Outcome Measure (HM-PRO): Content Validity
Source: Front Pharmacol. 2020 Mar 5;11:209. doi: 10.3389/fphar.2020.00209 (PMC7066982; doi:10.3389/fphar.2020.00209)
Supplement: Supplementary file 1 [file Data_Sheet_1.pdf]

Appendix I

No	First draft of HM-PRO used for Initial testing pre-CVM	Second draft of HM-PRO used for final testing post-CVM
**PART A**		
1	I am worried about biopsy	I am worried about biopsy/bone marrow test.
2	Transfusions are burden for me	Transfusions are a burden for me (e.g. blood, platelets).
3	I worry about my appearance	I worry about my appearance.
4	I feel distressed	I feel distressed.
5	I worry about money matters	I worry about money matters.
6	I have difficulty travelling	I have difficulty travelling (e.g. bus, train, flight and car).
7	I have difficulty going on holidays	I have difficulty going on holidays.
8	I have difficulty walking	I have difficulty with walking.
9	I have difficulty with self-care (e.g. dressing, bathing, etc.)	I have difficulty with self-care (e.g. dressing, bathing, etc.).
10	I have difficulty doing sports	I have difficulty with physical activity/sports.
11	I feel anxious	I feel anxious.
12	I worry about dying	I worry about dying.
13	I worry about treatment	I worry about treatment.
14	I have difficulty with personal relationships	I am having difficulty with personal relationships.
15	I worry about being burden to others	I worry about being a burden to others.
16	I am unable to socialize	I have difficulty socializing.
17	I have difficulty leaving the house	I have difficulty leaving the house.
18	I have difficulty with my work (or studies)	I have difficulty with work (or studies).
19	I have trouble with my appetite	I have trouble with my appetite.
20	I have difficulty managing my medication	I have difficulty managing my medication.
21	I have difficulty pursuing recreational activities and pastimes (e.g. gardening, art-work, etc.)	I have difficulty pursuing hobbies and pastime. (e.g. gardening, art-work, etc.).
22	I am worried about not having children	I am worried about not being able to have children.
23	My sleeping pattern has changed	My sleeping pattern has changed.

24	I don’t feel confident	I don’t feel confident.
25	I have difficulty doing daily activities	I have difficulty doing daily activities.
26	My eating habits have changed	My eating habits have changed.
27	My drinking habits have changed	My drinking habits have changed.
28	I am satisfied with the healthcare services	I am satisfied with the healthcare services.
29	I have problems with my sex life	I have problems with my sex life.
30	I am troubled with time spent in hospital	I dislike the time I spend in hospital.
31	I am worried about my future health	I am worried about my future health.
32	I am concerned about people judging me	I am concerned about people judging me.
33	I have difficulty concentrating	I have difficulty concentrating.
34		I am concerned about my weight change.
**PART B**		
1	I feel unwell	I feel unwell.
2	I feel tired	I feel tired.
3	I have difficulty breathing	I have difficulty breathing.
4	I have problem with my energy level	I have problems with my energy level.
5	I have back pain	I have back pain.
6	My weight has changed	*Item moved to PART A*
7	I have nausea	I have nausea.
8	I have body pain	I have body pain.
9	I have diarrhea	I have diarrhea.
10	I have hair loss	I have hair loss.
11	I have fever	I have/had fever.
12	I have night sweats	I have night sweats.
13	I have cough	I have/had a cough.
14	I have chest pain	I have/had chest pain.
15	I have lumps	I have lumps.
16	I have problems with my appetite	I have problems with my appetite.
17	I have stomach-ache	I have stomach ache.
18	I have headache	I have headaches.
19	I have skin problems (e.g. itching, bruises, rashes, etc.)	I have skin problems (e.g. itching, bruises, rashes, etc.).
20	I have constipation	I have constipation.
21	I have fatigue	I have fatigue.

22	I have infections (e.g. chest, lung, urinary, etc.)	I have infections (e.g. chest, lung, urinary, etc.).
23	I have vomiting	I have experienced vomiting.
24	I have problems with my bowel.	I have bowel problems.
25	I have problems with my taste	I have problem with my sense of taste.
